# Optimizing Recovery of High-Added-Value Compounds from Complex Food Matrices Using Multivariate Methods

**DOI:** 10.3390/antiox13121510

**Published:** 2024-12-11

**Authors:** Yixuan Liu, Basharat N. Dar, Hilal A. Makroo, Raouf Aslam, Francisco J. Martí-Quijal, Juan M. Castagnini, Jose Manuel Amigo, Francisco J. Barba

**Affiliations:** 1 Research Group in Innovative Technologies for Sustainable Food (ALISOST), Preventive Medicine and Public Health, Food Science, Toxicology and Forensic Medicine Department, Faculty of Pharmacy, Universitat de València, Avda. Vicent Andrés Estellés, s/n, 46100 Burjassot, Spain; yiliu2@alumni.uv.es (Y.L.); francisco.j.marti@uv.es (F.J.M.-Q.); 2 Department of Food Technology, Islamic University of Science and Technology, Awantipora 192122, Jammu & Kashmir, India; darnabi@iust.ac.in (B.N.D.); hilal.makroo@iust.ac.in (H.A.M.); 3 Department of Processing and Food Engineering, Punjab Agricultural University, Ludhiana 141004, Punjab, India; raoufaslam-pfe@pau.edu; 4 IKERBASQUE, Basque Society for the Promotion of Science, Plaza Euskadi, 5, 48009 Bilbao, Spain; josemanuel.amigo@ehu.eus; 5 Department of Analytical Chemistry, University of the Basque Country UPV/EHU, Barrio Sarriena S/N, 48940 Leioa, Spain

**Keywords:** optimization, high-added-value compounds, food industry, ANN, RSM

## Abstract

In today’s food industry, optimizing the recovery of high-value compounds is crucial for enhancing quality and yield. Multivariate methods like Response Surface Methodology (RSM) and Artificial Neural Networks (ANNs) play key roles in achieving this. This review compares their technical strengths and examines their sustainability impacts, highlighting how these methods support greener food processing by optimizing resources and reducing waste. RSM is valued for its structured approach to modeling complex processes, while ANNs excel in handling nonlinear relationships and large datasets. Combining RSM and ANNs offers a powerful, synergistic approach to improving predictive models, helping to preserve nutrients and extend shelf life. The review emphasizes the potential of RSM and ANNs to drive innovation and sustainability in the food industry, with further exploration needed for scalability and integration with emerging technologies.

## 1. Introduction

As science and technology continue to advance, consumer expectations for food product quality have reached new heights. This has prompted the exploration of artificial intelligence (AI) as a means to enhance predictive capabilities and meet the diverse needs of customers [1]. In tandem with scientific progress, technological advancements, and economic prosperity, there has been a growing emphasis on consumer health and food safety [2]. Previous research efforts have made significant contributions to food science. Prominent solutions encompass the utilization of response surface methodology (RSM) with both linear models and artificial neural networks (ANNs) [3]. These methods involve designing, modeling, and establishing quadratic equations to assess the responses of multiple variables, their quadratic terms, and their possible interactions. Within the extensive body of existing literature [1], there is growing interest in applying backpropagation in feedforward NNs for the optimization and modeling of various processes within the food industry.

RSM is a comprehensive toolkit that combines mathematical techniques and advanced statistics; thus, it holds a prominent position in both prediction and optimization. Its application involves a series of critical steps, encompassing the design of experiments, statistical analysis, and the optimization of variables, as outlined in detail elsewhere [4]. Researchers have predominantly relied on RSM to model complex multivariate processes, enabling the optimization of outcomes influenced by multiple variables [5]. It not only assesses the individual effects of independent variables but also accounts for their interactive responses. RSM, while useful for preliminary understanding, often relies on polynomial models that may lack physical or chemical significance, particularly when applied to factors like temperature or duration that might exhibit nonlinear responses. Therefore, RSM should be seen as a tool for initial exploration rather than a definitive optimization method. In the pursuit of achieving higher accuracy prediction models in agronomy and the food industry, a range of advanced techniques have been employed, including Partial Least Squares (PLSR), Gradient-Boosted Decision Trees (GBDT), K-Nearest Neighbors (KNN), and Random Forest (RF) [6]. Inspired by the biological neural systems of humans and guided by accumulated experiences, the ANN serves as a mathematical tool equipped with distributed, parallel, and adaptive data processing capabilities to model nonlinear systems in the pursuit of regression and/or classification/discrimination [7]. There are many architectures in ANNs, and feedforward backpropagation is one of the most common ones. A feedforward backpropagation ANN consists of three types of layers: an input layer, one or more hidden layers, and an output layer, each comprised of so-called neurons [8]. The versatile application of ANNs in food processing spans various domains, including food quality assessment, food extraction, food fermentation, food safety, food drying, and both thermal and non-thermal food processing operations [9]. In comparative studies evaluating prediction models for their overall acceptability in cooking rice, both linear modeling approaches (such as Multiple Linear Regression Analysis (MLR), Principal Component Regression, and PLSR) or other approaches more focused on a nonlinear modeling approach (ANNs, GBDT, KNN, and RF) were employed.

The computational functioning of ANNs closely emulates the biological operations of the human brain. This surge in interest arises from the pressing need for heightened accuracy and more comprehensive statistical capabilities compared to traditional food analysis methods [10]. The complementary combination of ANNs in the RSM umbrella processes serves a vital purpose in addressing the challenge of achieving higher extraction yields for high-added-value compounds. These methods excel in handling numerous variable nonlinear interactions, providing robust and over-determined interpretations, enhancing abstraction capabilities, mitigating interference, compensating for statistical deficiencies, and enhancing observational insights [11].

This combination emerges as a powerful and efficient tool for predicting and optimizing complex process models [12]. Moreover, it has been observed that ANNs consistently outperform RSM in the development of superior predictive models [13]. Building upon these insights, our present review was undertaken to predict and optimize responses in food processing.

Until now, there has been limited research that directly compares the optimization of high-added-value compounds’ recovery using RSM with an ANN. This gap in research is primarily due to the prevailing focus on exploring the optimal extraction conditions for independent variables and validating the resulting optimized parameters [14]. Various statistical parameters were employed to compare estimation capabilities and modeling performance. Notably, the coefficient of determination (R^2^) and root mean square error (RMSE) were among the critical metrics examined [14]. While both RSM and ANNs have been extensively studied independently, their combined application in optimizing the recovery of high-value compounds in the food industry remains underexplored. This study provides a novel approach by integrating these two methodologies, offering a more robust and accurate optimization framework. We provide a scalable optimization strategy that can be adapted to various food processing scenarios, potentially leading to significant improvements in yield and quality.

## 2. Materials and Methods Selection for RSM and ANNs

**Figure 1:**
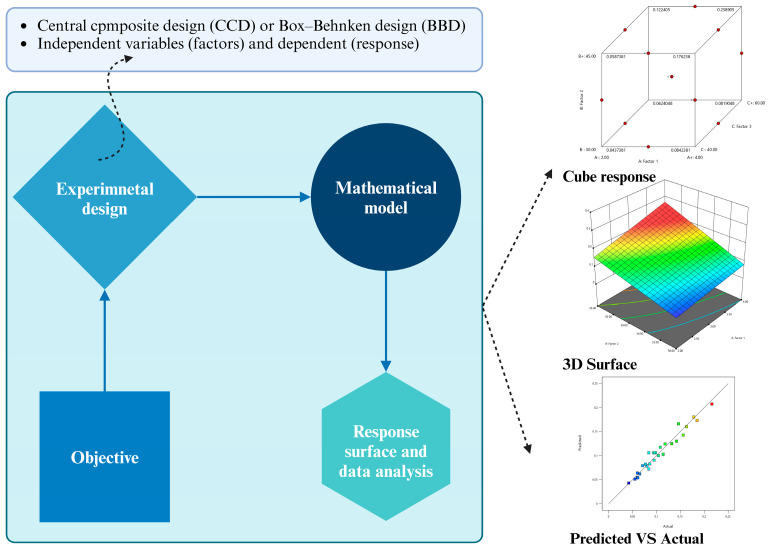
Flow chart of RSM (Created in BioRender.com).

Box and Wilson proposed RSM in 1951 [15], and it has since gained significant attention for its strong empirical performance in modeling. RSM is particularly effective in providing well-fitting models to describe the relationship between input parameters and responses. The flow chart of an RSM is illustrated in Figure 1. RSM employs two primary sampling methods: Box–Behnken Design (BBD) and Central Composite Design (CCD). BBD is a three-level design that avoids placing points at the vertices of a cubic region defined by the upper and lower limits of each variable. In contrast, CCD incorporates a full or fractional factorial design with center points, augmented by a group of axial points, which enables the estimation of the curvature in the model [16].

RSM is well-suited for modeling and optimizing linear and low-order nonlinear relationships, such as quadratic nonlinearity. However, its reliance on simple model assumptions, local approximation, and traditional experimental designs makes it less effective for complex nonlinear systems. For such cases, more advanced modeling approaches, such as artificial neural networks or machine learning models, are often necessary.

**Figure 2:**
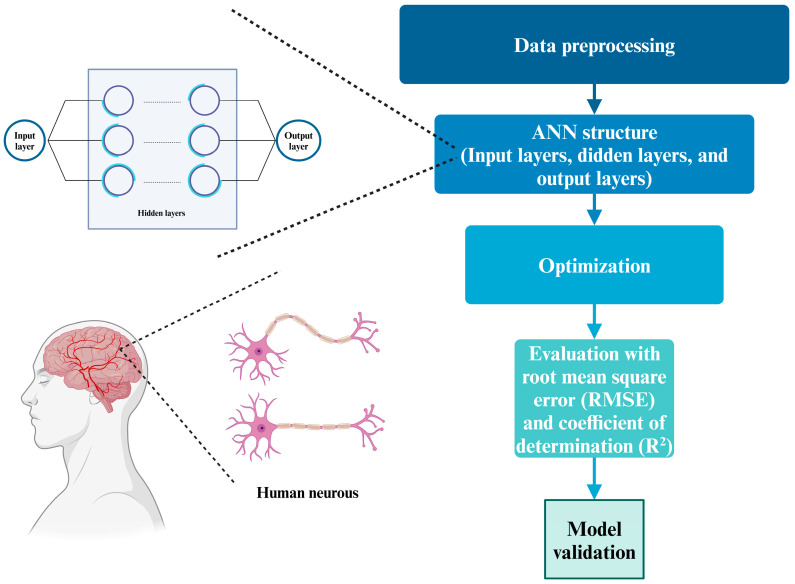
Flow chart of ANN (Created in BioRender.com).

Artificial Neural Networks (ANNs) are a mathematical modeling approach that utilize neural network structures to simulate physical systems [17]. The foundational theory of ANNs was first introduced by McCulloch and Pitts in 1943 [18]. The flow chart of an ANNs is illustrated in Figure 2. ANNs are simplified representations of biological nervous systems, capable of mapping nonlinear relationships and predicting outcomes even with incomplete data. Through the use of connection weights that link inputs, hidden layers, and outputs, ANNs achieve accurate predictions. The parallel architecture of ANNs ensures rapid responses and low computation times, making them particularly suitable for real-time food processing applications [19]. A commonly employed supervised learning algorithm for training multilayer perceptron (MLP) neural networks in solving nonlinear food-engineering problems is the backpropagation algorithm [20]. Consequently, ANNs are highly recommended for modeling nonlinear systems in food engineering.

A recent study simplifies the understanding of the materials and methods for RSM and ANNs by investigating a hybrid RSM–ANN–Genetic Algorithm (GA) approach to optimize extraction conditions for bioactive component-rich laver (*Porphyra dentata*) extracts [4]. This research compares and optimizes infusion extraction and ultrasound-assisted extraction using both RSM and the hybrid RSM–ANN–GA methods. The optimization focused on the total phenolic, flavonoid, and amino acid contents, as well as the color value and R-phycoerythrin content. The experimental variables included temperatures (60, 80, and 100 °C) and times (10, 15, and 20 min), and were designed to optimize these parameters in the laver extract. Modeling began with RSM using a three-level factorial design, where the response variables were analyzed based on experimental runs generated by the RSM model. Subsequently, the experimental data from the RSM design points were used to develop the ANN–GA model, identifying the optimal neural network architecture. The extraction conditions were then optimized using statistical analyses based on the RSM and ANN–GA calculations. Similarly, a recent study demonstrated that ANN–GA predictions yield a higher accuracy and align more closely with experimental data compared to RSM-predicted values [5].

## 3. Optimization Based on Nutrients (Carbohydrates, Proteins, Lipids, and Fatty Acids)

### 3.1. Carbohydrates

**Figure 3:**
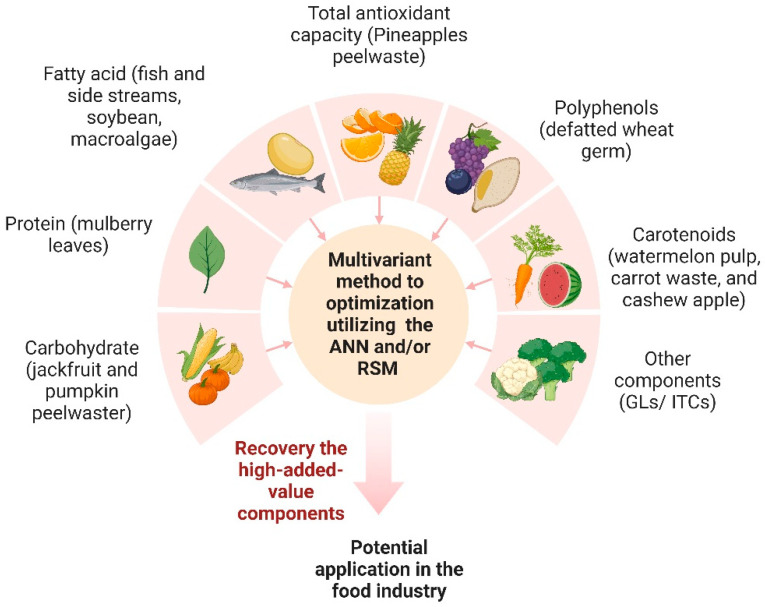
Recovery of high-added-value components from the food industry using the ANNs or RSM optimization method (Created with BioRender.com).

Carbohydrates, being natural organic molecules, primarily consist of carbon, hydrogen, and oxygen, and play a pivotal role in the human body due to their biocompatibility and biodegradability [21]. A better understanding of the relationship between nutrient recovery and ANNs or RSM is summarized in Figure 3. Consequently, carbohydrates and polysaccharides offer several advantages, including their chemical stability, resistance to protein interactions, well-defined chemical structures, ease of formation, water solubility, and compatibility with various cell receptors. These attributes make them highly proficient components in delivery systems [21]. Polysaccharides, widely found among animals, microorganisms, and plants, are well-recognized for their diverse resources [22]. These macromolecules exhibit significant variability in their chemical structures, influenced by factors such as the types of monosaccharide units, their linkages, molecular weights, and the presence of other substituents. Polysaccharides, characterized by their glycosidic bonds linking monosaccharide units, typically possess higher molecular weights, along with diverse structural arrangements. These attributes, like their monosaccharide compositions, linkages below sugar residues, and solution conformations, contribute to their pivotal roles in numerous physiological activities [23]. In recent years, there has been a growing focus on exploring the biological activities of polysaccharides. These include their immunomodulatory and anti-diabetic properties [22], as well as their anti-tumor, antioxidant, hypotensive, cardioprotective, and prebiotic activities [24]. Furthermore, polysaccharides have found applications in biomaterials, biofuels, and pharmaceuticals over the past few decades [25].

Starch, one of the most abundant polysaccharides, finds global utility in the food industry. RSM with an ANN has been employed to enhance starch extraction processes. Following a comparative analysis, it was found that the RSM exhibited superior efficiency, as evidenced by a higher R² and lower average absolute deviation (AAD) during both the design and validation stages [26]. This resulted in a remarkable improvement in the starch yield from *Amorphophallus paeoniifolius* corms, with a yield of 51.76 g/100 g of corm dry weight, representing an increase of 7.61–27.24-fold over previous methods [26]. In another application, the ultrasound-assisted extraction of *Ginkgo biloba* leaves polysaccharides (GBLPs) underwent optimization using RSM, resulting in an optimized GBLP yield of 5.37%. This was achieved with the ultrasonic power set at 340 W, a liquid-to-material ratio of 30 mL/g, and an extraction time of 50 min [27]. Pectin, a naturally occurring biopolymer, holds significant importance in biotechnology and the food industry. It is known for its contributions to enhancing stability, as well as its role as a carrier for protecting the targeted delivery of bioactive compounds [28]. Recent research efforts combined pulsed electric fields (PEFs) with microwave-assisted extraction for pectin polysaccharide extraction from jackfruit waste. This process was optimized and analyzed using both Box–Behnken Design (BBD) and an ANN. The optimized treatment conditions obtained by BBD included 11.99 kV/cm of PEF strength, 4.00 min of PEF treatment time, 647.30 W/g of MAE power density, and 5.00 min of MAE exposure time. Notably, the ANN (R^2^ between 0.95 and 0.99, and mean squared error between 0.008 and 0.1) model outperformed the BBD (R^2^ between 0.89 and 0.98, and sum of squared error between 0.076 and 0.781) in terms of performance and accuracy [29].

### 3.2. Proteins

Protein, a vital macronutrient, is witnessing a rapid shift in demand for new sources. Notably, nutrient deficiencies in dairy diets have affected over two billion people, particularly in developing and underdeveloped countries facing challenging circumstances due to such deficiencies [30].

Traditional methods for vegetable protein extraction typically involve alkaline extraction. This process enhances protein solubility in aqueous solutions by disrupting the hydrogen bonds and unfolding the protein matrix under alkaline conditions [3]. However, challenges arise when proteins are bound with substances like cellulose in green-leaved plants, limiting the protein extraction yield [31]. Extraction processes assisted by various novel methods hold significant potential for improving the yield of target products. Ultrasound, for instance, has found extensive applications in food physical processing, particularly for the extraction of macromolecular substances, including proteins [32]. Ultrasound primarily modifies proteins by disrupting water and hydrogen bonds through ultrasonic cavitation and mechanical effects. It also induces alterations in secondary protein structures, leading to increased protein solubility by enhancing protein repulsion [33].

The ultrasound-assisted cellulase degradation (UACD) method was used to extract mulberry leaf protein, and the process parameters were optimized to maximize the protein dissolution amount (PDA) from mulberry leaves. This optimization process combined single-factor experimentation and RSM [34]. Fourier transform infrared spectroscopy (FTIR) and scanning electron microscopy (SEM) were employed by previous researchers to analyze the extracted mulberry leaf protein. The results demonstrated that under the optimized conditions (a pH value of 7.2, an ultrasound temperature of 35.0 °C with an enzyme dosage of 4.2%, and an ultrasound time of 10.0 min), the experimentally verified PDA value was 13.87 mg/mL, closely aligning with the predicted value of 13.5 mg/mL [34].

### 3.3. Lipids and Fatty Acids

Previous studies have collectively provided valuable insights into lipid oxidation, which encompasses autoxidation, thermal oxidation, enzymatic oxidation, and photo-oxidation. These processes typically involve the utilization of free radicals or reactive species as intermediates. A better understanding of the relationship between nutrient recovery and ANNs or RSM is summarized in Table 1.

In a prior study, lipids were comprehensively classified and defined, resulting in their categorization into eight representative groups. These include fatty acids (FAs), glycerolipids (GLs), glycerophospholipids (GPs), sphingolipids (SPs), sterol lipids (STs), prenol lipids (PRs), saccharolipids (SLs), and polyketides (PKs). The classification of lipids has expanded to encompass over 47,000 variations, posing significant challenges in the analysis of these diverse lipid types and complex lipid structures [35]. In general, oils extracted from sources such as fish, shellfish, microalgae, and plants are characterized by exceptionally high levels of polyunsaturated fatty acids, rendering them prone to instability [36]. Lipid oxidation, as elucidated in prior studies, is a multifaceted process. Unsaturated fatty acids generate unstable products during reactions, which subsequently decompose and polymerize into secondary products [36]. Furthermore, to enhance the mechanical and barrier properties of soybean aqueous extract-based films, process parameters were optimized using RSM, and film properties were predicted using an ANN. The resulting film exhibited outstanding antioxidant properties and a superior morphology [37]. The ANN approach was also employed to determine the optimum culture conditions for biodiesel production. This optimization yielded optimal conditions, including a pH of 7 after 16 days of cultivation, resulting in total lipid, unsaturated lipid, and biomass concentrations of 1266.33 mg/L, 1072.058 mg/L, and 2663.34 mg/L, respectively [38].

**Table 1:** Nutrient recovery from food processing using ANN or RSM as multifactorial decision tools for optimization.

Target Compounds Are Taken Into Account	Type of Multifactorial Design	Source	Optimal Conditions	Reference
Bioactive polysaccharides	RSM; the genetic algorithm–artificial neural networks (GA–ANN)	*Bletilla striata*	52 °C, 167 min, and 0.01 mol/L NaOH (alkali-assisted extraction)	[39]
Carbohydrate	RSM	Folium Ginkgo	Water/solid ratio of 4, extraction of 95 °C for 120 min	[40]
Maximum reducing sugar	ANN; RSM	Pumpkin peel waster	Hydrolysis time of 120 min, loading substrate of 17.5 g/L, α-amylase concentration of 7.5 U/g, amylglucosidase concentration of 56.40 U/mL, fermentation temperature of 45 °C, pH of 5.06, shaking speed of 188.5 rpm, and yeast concentration of 1.95 g/L	[41]
Polysaccharides	RSM	Zagros oak leaf	Ultrasonic power of 205.8 W, extraction temperature of 81.9 °C, extraction time of 55.6 min, and ratio of water to raw material of 23.4 (ultrasonic-assisted extraction).	[42]
The optimization of microwave-assisted phosphorylation modification of MBP and its effect on functional and structural characterizations	RSM	Mung bean protein (MBP)	STP/protein ratio of 0.063 g/g, microwave power of 590 W, microwave time of 155 s, and pH of 7.8.	[43]
Higher biomass productivity, lipid content, and productivity	RSM	Dunaliella parva (marine microalga)	Optimized medium containing 0.63 g/L of nitrogen, 0.02 g/L of phosphorus, and 1.61 M of NaCl	[44]
Algal lipids	RSM	Marine macroalgae	Extraction temperature of 72 °C, solvent-to-algae ratio of 5:1, algal particle size of 0.16 mm, and extraction time of 134 min.	[45]
Methyl esters yield from Anacardium occidentale kernel oil	RSM and ANN	Anacardium occidentale kernel oil	At modeling conditions, including temperature of 65 °C, mole ratio of 7:1, catalyst concentration of 2.5 wt %, stirring speed of 600 rpm, and time of 150 min, RSM-predicted and validated methyl ester yields were 94.82%, and 94.70%, respectively. In contrast, ANN-predicted and validated yields were 93.21% and 93.33%, respectively.	[46]
n-3 Long-chain polyunsaturated fatty acids (LC-PUFAs)	RSM	Tuna oil	Optimal conditions for hydrolysis reactions were reaction temperature of 54 °C, enzyme load of 3%, reaction time of 6 h, and mass ratio of water to tuna oil of 1.5:1.	[47]
Higher retention of bioactive compounds	Artificial neural network–particle swarm optimization (ANN–PSO) and RSM	Finger mille (*Eleusine coracana*)	ANN–PSO modeling outperformed RSM in process prediction, and optimization conditions for ANN–PSO were a 76% amplitude, water-to-grain ratio (W:G) of 3.5:1, and 17.5 min treatment time at 40 kHz.	[48]
Monitoring the lipid oxidation and fatty acid profile of oil using algorithm-assisted surface-enhanced Raman spectroscopy	ANN and principal component analysis (PCA)	Sunflower oil (SO)	Developed Raman and SERS methods could detect low levels of 2% trans fats with a sensitivity of 94% and 97%, respectively.	[49]
Fatty acid and tocopherol content	ANN and random forest regression (RFR)	Rapeseed (*Brassica napus* L.) oil	RFR and ANNs are prosperous for modelling rapeseed seed quality, and the ANN model predicted erucic acid with highest accuracy.	[50]

## 4. Optimization Based on Antioxidant Response (Total Antioxidant Capacity, Polyphenols, Carotenoids)

### 4.1. Total Antioxidant Capacity (TAC)

It is widely accepted that natural antioxidants, including carotenoids (such as carotenes and xanthophylls), polyphenols (including flavonoids, phenolic acids, and anthocyanins), and vitamins (such as vitamin C and E), play a pivotal role in various biological functions. Notably, these compounds exhibit properties such as antifungal, antibacterial, and anti-inflammatory effects [51]. The antioxidant capacity of natural compounds can be evaluated based on five key characteristics: their reduction potential, the fate of the resultant antioxidant-derived radical compound, their capacity for the stabilization and delocalization of unpaired electrons, their reactivity with other antioxidant compounds, and their potential for chelating transition metals. These factors collectively determine their antioxidant effectiveness [52]. Previously published peer-reviewed research data have laid the foundation for assessing the antioxidant potential of natural products. This assessment forms the basis for recommendations regarding the best antioxidant-rich foods and the ranking of antioxidant-rich plants. Contrary to becoming reactive free radicals, antioxidants mitigate reactivity by donating electrons to free radicals, a process considered beneficial for human health. The antioxidant activity can be quantified using assays such as the oxygen radical absorbance capacity (ORAC) assay. This method relies on the changes in fluorescence during the reaction between fluorescein sodium and peroxyl radicals (ROO˙), with a decrease in fluorescence indicating the extent of antioxidant activity [53].

A comparative assessment of the performance of the ANN and RSM was conducted to optimize microwave-assisted extraction (MAE) and evaluate the antioxidant capacity of extracts obtained from *Vernonia cinerea* leaves. The ANN models exhibited superior performances, as evidenced by higher R², lower RMSE, and absolute average deviation (AAD) values. It is worth noting that both the ANN (0.9912 for yield, 0.9928 for DPPH, 0.9928 for DPPH, and 0.9944 for ABTS in R^2^, respectively) and RSM (0.9590 for yield, 0.9888 for DPPH, 0.9891 for DPPH, and 0.9944 for ABTS in R^2^, respectively) demonstrated commendable estimation capabilities [54]. Similarly, the impact of ultrasound-assisted extraction (UAE) parameters, including the extraction time, acetone concentration, and solvent-to-solid ratio, on kidney bean extraction was investigated and optimized using RSM and ANNs (ANN–radial basis function (RBF) and ANN–multi-layer perception (MLP)). The optimization process resulted in the attainment of maximum values for two output parameters, namely, 917.2 mg GAE/100 g DW of total phenolic content (TPC) and 56.03 μmol Trolox/g DW of ABTS (2,2′-azino-bis (3-ethylbenzothiazoline-6-sulfonic acid)) antioxidant activity. These optimal conditions were determined to be a solvent-to-solid ratio of 37 mL/g, an acetone concentration of 53%, and an extraction time of 70 min [55]. Furthermore, it is noteworthy that the ANN model outperformed the RSM model in optimizing kidney bean antioxidants, with estimative values demonstrating a hierarchy of prediction accuracy: ANN–RBF (TPC: 0.9950 for R^2^, 8.530 for RMSE, and 0.436 for AAD (%); and ABTS: 0.9885 for R^2^, 1.010 for RMSE, and 0.119 for AAD (%) > ANN–MLP (TPC: 0.9851 for R^2^, 14.82 for RMSE, and 1.499 for AAD (%); and ABTS: 0.9764 for R^2^, 1.195 for RMSE, and 0.139 for AAD (%) > RSM (TPC: 0.9791 for R^2^, 17.10 for RMSE, and 1.862 for AAD (%); and ABTS: 0.9666 for R^2^, 1.275 for RMSE, and 0.153 for AAD (%).

### 4.2. Polyphenols

Non-enzymatic antioxidants, including phenolics, tocopherols, carotenoids, and vitamin C, alongside the body’s enzymatic systems, such as catalase, superoxide dismutase, and glutathione peroxidase, collectively safeguard the human body against oxidative damage. Numerous studies have explored the close association between the total phenolic content (or individual phenolics) and overall antioxidant activity [56]. The research presented thus far offers compelling evidence that polyphenols derived from grains have a beneficial impact on the proliferation of beneficial intestinal bacteria. Moreover, plant polyphenols have demonstrated their potential as quorum-sensing inhibitors, effectively regulating the expression of virulence factors in harmful intestinal bacteria [57].

Polyphenols are characterized by the presence of one or more aromatic rings linked to one or more hydroxyl groups. These compounds are produced as secondary metabolites in plants, boasting a diverse array of over 800 structures, which include phenolic acids, flavonoids, tannins, and stilbenes [58]. Polyphenols exhibit a wide range of functionalities, encompassing antioxidant properties, anticancer activities, and anti-inflammatory effects. Furthermore, they can modulate transcription factors such as Nrf-2 and NF-kB, thereby amplifying their bioactive effects and mitigating oxidative stress [59].

Due to the intricate nature of phenolic compounds, the efficacy of complete extraction processes is influenced by the preparation methods. As a result, there is no universally accepted protocol for phenolic extraction processes [60]. The studies presented thus far underscore the importance of establishing an optimization process for phenolic extraction. This optimization should take into account factors such as the availability of techniques, the specific analysis objectives, the targeted compounds, and the nature of the sample, all of which should be considered comprehensively [60].

Previous research has harnessed the RSM to optimize the extraction of polyphenolic compounds from tomatoes, successfully predicting the optimal extraction conditions [61]. Similarly, RSM design and analyses were employed in the investigation of ultrasound-assisted extraction (UAE) for antioxidant compounds, total phenolics, and soluble proteins from bitter gourd (*Momordica charantia*) [62]. The pulsed-mode sonication approach yielded optimal bioactive compound extraction, resulting in an antioxidant activity of 77.9%, a total soluble protein content of 42.1 mg/1000 mL, and a total polyphenol content of 104.5 mg GAE/g. These optimal conditions included an extraction time of 12 min, a temperature of 68.4 °C, and a bitter gourd to water ratio of 0.25 g/mL. To assess the predictive capabilities of the developed models from RSM and ANN, various statistical parameters, including R^2^, AAD, and RMSE, were considered regarding phenolic compounds and antioxidant activity [63]. In a separate study, the correlation between RSM and ANNs in predicting bioactive compounds from unripe *Musa acuminata* peel was explored. The ANN model demonstrated a superior predictive potential, as indicated by its higher R^2^ (R^2^_RSM_ = 0.9742, R^2^_ANN_ = 0.9803), lower AAD (AAD_RSM_ = 5.64%, AAD_ANN_ = 3.38%), and lower RMSE (DMSE_RSM_ = 3.07, DMSE_ANN_ = 2.69) [63].

### 4.3. Carotenoids

Carotenoids are recognized as pigments accumulated in the chloroplasts and chromoplasts of various organisms, including plants, bacteria, fungi, photosynthetic algae, animals, and shellfish [64]. These lipid-soluble tetraterpenoids consist of 40 carbon atoms with multiple double bonds and are predominantly categorized into two groups: carotenes. Notable examples include β-carotene and lycopene [64]. Carotenoids play pivotal roles in the physiology of animals, encompassing functions such as cancer prevention, immune system modulation, regulation of growth factors and intracellular signaling pathways, control of metabolic activity in cells, offering photoprotection against UV radiation, and serving as precursors for the visual pigment retinol.

The optimization of microwave-assisted carotenoid extraction was achieved through the application of RSM. The study focused on determining the ideal microwave power, extraction time, and oil-to-waste ratio for maximizing carotenoid recovery. The optimized conditions were identified as 165 W of microwave power, 9.39 min of extraction time, and an oil-to-waste ratio of 8.06:1 g/g, resulting in a carotenoid recovery rate of 77.48% [65]. In another investigation, the valorization of waste apricot flesh (WAF) was explored, with the extraction conditions optimized using the Box–Behnken design. This approach led to the attainment of a total carotenoid content (TCC) of 42.75 mg/100 g of dried weight under the following optimized conditions: 60 min of extraction time, a temperature of 41.53 °C, 200 W of power, and a liquid-to-solid ratio of 0.10 g/mL [66]. The comparison of the extraction yield of chlorophylls and carotenoids from the wet and heat-dried isolated microalgal (*Chlorella thermophila*) biomass using ethanol has been optimized with ANN, and the result found that chlorophyll extraction yield was observed to be 2.7 fold higher from wet biomass than dry biomass while carotenoid yield was 6.7 fold higher and the highest chlorophyll yield (∼60 mg/g-dry biomass) was observed at 6 min of homogenization time, 10,000 rpm, 1 mg/mL of solid solvent ratio, and 58 °C of solvent temperature from wet biomass with extraction efficiency of approximately 94% [67].

While certain methods have been developed for carotenoid extraction, they are often associated with drawbacks such as high costs, extended extraction times, and the use of organic solvents, which can impose limitations on the efficiency of carotenoid extraction [68]. In this context, the valorization of food side streams offers a promising solution to address both environmental and economic challenges while meeting the demand for natural carotenoids. A better understanding of the relationship between the antioxidant compounds of the ANN and RSM is summarized in Table 2.

## 5. Optimization Based on Other Compounds (Glucosinolates (GLSs)/Isothiocyanates (ITCs)

Glucosinolates (GLSs), well-recognized as a significant group of sulfur-rich anionic secondary metabolites, are prevalent in plants belonging to the *Brassicaceae* family [78,79,80]. Among these plants, the *Brassicaceae* family is renowned for having the highest concentrations of GLSs, with examples including horseradish, broccoli, wasabi, and various other species [81]. Prior studies have delved into optimizing the hydrolysis of GLSs found in Brassica vegetables to produce isothiocyanates [82]. Furthermore, efforts have been made to enhance the myrosinase activity sourced from select Brassica vegetable varieties. In this regard, an optimal myrosinase extract was achieved to facilitate the production of sulforaphane from glucoraphanin. Notably, the highest myrosinase activity was attained under specific conditions, including a vacuum pressure of 40 mbar and a temperature of 40 °C, with a duration of 90 min, as identified in this research [83].

Previous studies collectively offer valuable insights into the fermentation products generated through microbial activity, specifically the conversion of sugar into lactic acid. Cruciferous vegetables have a long history of being utilized as fermented products, and the complete fermentation of GLSs occurs during this process [81]. A comparison between ANNs and RSM for optimizing fermentation media in the production of the biopolymer scleroglucan has been explored. The yield of scleroglucan (g/L) was modeled and optimized based on four independent variables, namely sucrose, yeast extract, K_2_HPO_4_, and MgSO_4_ concentrations (g/L). The ANN approach demonstrated superior accuracy, with an average error of 6.5% compared to 20.0% for RSM. Furthermore, the correlation coefficient values were 0.98 for the ANN and 0.89 for RSM, highlighting the enhanced precision of the ANN in this context [84]. Additionally, the degradation products of GLSs possess bioactive properties. These hydrolysis products encompass isothiocyanates (ITCs), thiocyanates, oxazolidinethiones, epithionitriles, and nitriles, each exhibiting distinct bioactive attributes [85,86].

The hydrolysis of sinalbin to produce 4-hydroxy benzyl-isothiocyanate (4-OH-ITC) from yellow mustard seeds (*Sinapis alba*) was systematically optimized based on myrosinase activity utilizing RSM. The highest hydrolysis conversion of sinalbin to 4-OH-ITC, reaching 20.1 mg/g of seeds, was achieved under optimal hydrolysis conditions. These conditions included homogenization for 5 min at a pH of 5.8, adding 4.5 mM of ascorbic acid, and incubating at 51.0 °C for approximately 15.8 min [87]. RSM, with its establishment of mathematical models and consideration of variable parameters, emerged as an efficient optimization methodology for this process. In another application of RSM, the optimization of folate extraction from date palm fruits involved enzymatic de-pectinization. The highest extraction yield was attained under optimized conditions, which included 47.7 U of pectinase, an incubation temperature of 40 °C, and an incubation time of 38 min [88].

The optimization of the fermentation process for xylitol production from areca nut husk involved the utilization of both ANNs and RSM. This optimization considered five variable parameters: agitation (rpm), pH, temperature (°C), time (h), and xylose concentration (g/L). The outcomes from the ANN model demonstrated a significantly higher level of robustness and precision in estimating the dependent variables when compared to the RSM model, and the maximum concentration of xylitol achieved was approximately 9.96 g/L under the designed conditions [89]. The studies conducted thus far shed light on the enhanced accuracy in modeling and prediction offered by ANN models when compared with the use of RSM models. Researchers have attributed this phenomenon to the ANN’s capacity for sensitivity analysis and the absence of a need for the prior specification of a suitable fitting function. In contrast, RSM is structured and valuable for estimating the likelihood of interactions among different components [89].

## 6. Application for Conventional and Innovative Extraction Approaches

### 6.1. Conventional Extraction Approaches

Traditionally, the extraction technique often deviates from the ideal, resulting in the use of conventional extraction programs that rely on a multitude of solvents and, at times, manual procedures that vary depending on the investigator and company’s preferences [90]. To illustrate this, when extracting polyphenols, one can choose from a range of conventional methods such as Soxhlet extraction, maceration, infusion, serial exhaustive extraction, percolation, and decoction [91]. Typically, organic solvents like water, methanol, n-hexane, ethanol, acetone, chloroform, propanol, and ethyl acetate are employed due to their ease of mixing and high extraction yields.

Normally, the technique is not consistent with the ideal, as a consequence of the fact that the traditional extraction program is designed with a large number of solvents and some accidental manual procedures depend on the investigator and the company decision maker [90]. As an illustration, in the extraction of polyphenols, there are various choices for conventional methods, including Soxhlet extraction, maceration, infusion, serial exhaustive extraction, percolation, and decoction [91]. Generally, water, methanol, n-hexane, ethanol, acetone, chloroform, propanol, and ethyl acetate are used as the solvent for extracting, as those organic solvents have the advantage of easily mixing easily and have a high extraction yield.

A previous research article has identified several conventional plant extraction methods, including maceration extraction (ME), infusion, digestion, decoction, percolation, and Soxhlet extraction [92]. Over the last few decades, it has become widely acknowledged that these traditional extraction techniques offer high yields without requiring complex instrumentation, such as magnetic stirring extraction or advanced maceration and Soxhlet extraction methods [93]. However, it is important to recognize that there are significant drawbacks associated with prolonged extraction times, substantial organic solvent usage, and potential environmental impacts [93]. As a result, there is a growing need for more sustainable and environmentally friendly extraction practices. While the extraction of high-added-value compounds using organic solvents showcases remarkable efficiency without the need for advanced equipment, its application in food products is constrained by the challenges of its high costs, potential toxicity, and the risk of residual contamination in the final products.

### 6.2. Innovation Processing Extraction Approaches

These novel extraction technologies have great potential for optimized extractions in the food industry. For instance, previous studies have demonstrated that subcritical water (SW) has a miraculous characteristic where it can remain in the liquid state under the boiling-point temperature (100.0 °C) and the critical-point temperature (374.0 °C) [94]. The SWE technology has the advantage of being chemical-free, as well as having shorter times and lower costs, and is characterized by associated advancements in extracting bioactive compounds [95]. Additionally, supercritical fluid extraction (SFE) exhibits an extraordinary solvating capability akin to hexane. This property finds applications in the recovery of high-purity, thermo-labile compounds, which are exemplified by carotenoid extracts [96].

Despite the existence of various parameters or independent variables, such as the solvent composition, extraction temperature, solvent-to-solid ratio, and particle size, which can influence the extraction process and its dependent variables, the predicted responses obtained from the ANN model demonstrate a high level of accuracy. This is evidenced by a lower relative deviation and RMSE, as well as a higher R^2^ value [19]. The utilization of conventional methods for extracting high-value compounds from foods and food by-products presents several drawbacks. Some of the most common issues include a low extraction efficiency, extended extraction times, chemical consumption, and environmental hazards. However, innovative and eco-friendly technologies have emerged to overcome these limitations. These include enzyme-assisted extraction (EAE), pulsed electric field (PEF), SFE, accelerated solvent extraction (ASE), high-pressure processing (HPP), ultrasound-assisted extraction (UAE), microwave-assisted extraction (MAE), subcritical water extraction (SWE), and pressurized liquid extraction (PLE) [95]. As an example of the application of such innovative techniques, UAE was employed to recover phytocompounds from dragon fruit peel, and the optimization process combined an ANN with genetic algorithms (GAs). The optimized conditions included an ultrasonic temperature of 60.0 °C, a solvent-to-sample ratio of 25:1 mL/g, a solvent concentration of 60%, and an ultrasonic treatment time of 20.0 min. These conditions are suitable for industrial implementation and offer accurate predictions and estimations for operational purposes [97].

**Figure 4:**
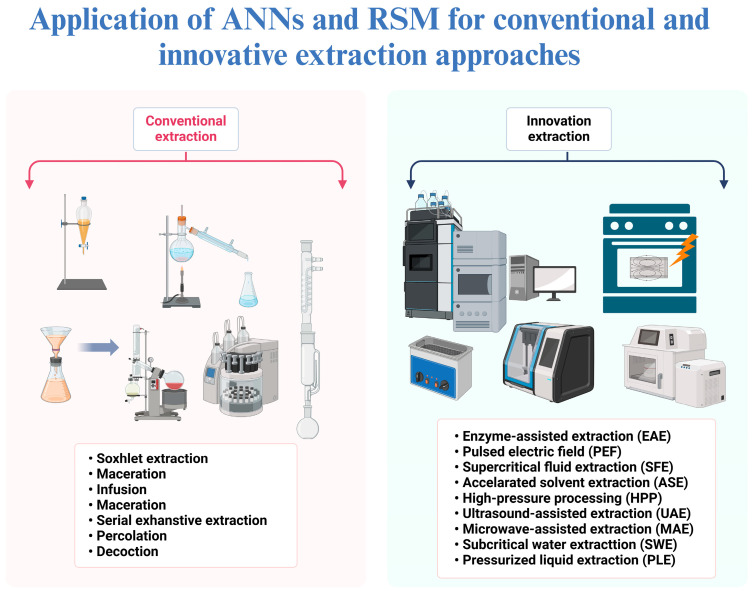
Application of Artificial Neural Networks (ANNs) and Response Surface Methodology (RSM) for conventional and innovative extraction approaches (Created with BioRender.com).

While both RSM and ANNs offer specific advantages in terms of structured experimentation and handling complex datasets, other methods, such as genetic algorithms, may provide more flexibility in exploring a wider parameter space without relying on predetermined models. A comparative analysis suggests that a hybrid approach, integrating these different methods, may offer the most robust solutions for optimizing food processing operations. A better understanding of the application of ANNs and RSM for conventional and innovative extraction approaches is illustrated in Figure 4.

## 7. Advantages and Drawbacks

### 7.1. Advantages

ANNs offer a distinct advantage by exhaustively exploring all potential scenarios and selecting optimal parameters, eliminating the need for repetitive experimentation when testing new processing conditions [19]. In contrast to many traditional chemometric methods that struggle to accommodate nonlinear trends in data, ANNs excel in effectively addressing and navigating these nonlinear patterns in experimental data, consistently yielding highly favorable results [19,98]. This adaptability sets ANNs apart from other modeling approaches. When compared to conventional regression or classification analyses, ANNs demonstrate remarkable precision in predicting response variables, optimizing processes, and requiring fewer experimental trials to achieve reliable outcomes [54,99].

In the realm of food processing, while RSM has historically been a widely adopted modeling tool, ANNs have emerged as an alternative method with superior performance, exceptional predictive capabilities, and the ability to model complex nonlinear problems [100]. While RSM and ANNs have been widely used for optimizing various extraction processes, a critical analysis reveals that the effectiveness of these methods can vary significantly depending on the context, such as the type of raw material, solvent, and processing conditions. The reviewed studies demonstrate a consistent trend, with RSM providing a structured approach for initial experimentation, whereas ANNs excel in handling more complex, nonlinear relationships.

### 7.2. Drawbacks

It is widely acknowledged that certain key parameters, namely temperature, pH, solvent selection, and duration, hold significant importance in conventional extraction processes [101]. In the context of protein extraction, suboptimal heating conditions can lead to excessive protein denaturation, resulting in proteins with compromised functional and nutritional properties. Similarly, high pH conditions may induce multiple protein aggregations, further affecting the quality of the extracted proteins [102].

While the SC-CO_2_ technique eliminates the need for chemical solvents in carotenoid extraction, it has been observed that this method tends to recover more chlorophylls than carotenoids, ultimately impacting the purity of the extract [103]. Remarkably, recent research has highlighted a significant drawback of ANN modeling: the “black box” nature of the model, which makes it challenging to discern the exact reasons behind specific outputs [19]. Furthermore, it is worth mentioning that ANNs consume a multitude of statistics, potentially numbering in the thousands or millions, during the overfitting process, setting them apart from traditional machine learning algorithms [19]. Many of the studies reviewed have limitations related to their specificity with respect to local conditions, such as the particular raw materials, solvents, and equipment in the studies, which may not be directly transferable to other contexts. This raises concerns about the generalizability of the findings, highlighting the need for further research to validate these optimization methods across different scenarios.

## 8. Potential Scale-Up and Industrial Applications

### 8.1. Potential Application of RSM and ANNs in the Food Process Industry

Some attempts have been made to introduce mechanization into the continuous food industry. In one such endeavor, the Box–Behnken (BBD) design and ANNs were employed to optimize the cocoa pod breaking and bean extraction process across various parameters. The results indicate the superior performance of the ANN design over the BBD approach [11]. Under the optimized conditions, which included a roller speed of 360 rpm, a pod size of 160 mm, and an inclination angle of 45°, remarkable outcomes were achieved. These included a maximal capacity (636.8 kg/h), outstanding beans separation efficiency (86.4%), high shelling efficiency (96.3%), excellent machine efficiency (96.78%), minimal energy consumption (10.54 kJ), and a negligible percentage of beans damaged (<1%) [11].

ANNs have found applications in various aspects of food processing [1], including drying, extraction, separation techniques, extrusion, fermentation, high-pressure processing (HPP), and power technology. Moreover, ANNs have been effectively employed to assess and predict food quality and properties, encompassing their chemical composition, nutrient analysis, texture and rheology, color evaluation, thermal conductivity measurement, classification, and quality control, as extensively documented in prior research [1].

An experimental and theoretical investigation was conducted to study shelled corn drying in a microwave-assisted fluidized bed dryer. The study involved four levels of air temperature (30.0, 40.0, 50.0, and 60.0 °C) and six levels of microwave power (0, 180.0, 360.0, 540.0, 720.0, and 900.0 W) using a ANN for prediction. The research also assessed the impact of uncertainties in output experimental data and ANN prediction values on the RMSE, which resulted from minor random errors within a range of ±5.0% [104]. In another study, plum pomace lyophilizate (PPL) at concentrations of 2.0–10.0%, sugar (sucrose) at concentrations of 10.0–15.0%, and amidated low-methoxy pectin (LMA) at concentrations of 0–0.2% were considered as independent variables for the optimization of product formulations. The optimal quality parameters were achieved with 10.0% PPL, 15.0% sucrose, and 0.0% LMA [105]. The findings from these studies have significant implications for the food industry. By optimizing the recovery of high-value compounds through the integrated use of RSM and ANNs, these studies not only enhance efficiency and yield but also contribute to more sustainable processing practices. These advancements align with the growing demand for environmentally responsible food production methods.

The optimization methods discussed in this review, while useful for practical applications, also provide valuable insights for food process engineering. The ability to optimize extraction processes is not only relevant to production efficiency but also to the design of new processes and equipment, making these findings applicable to both academic research and industrial practice.

### 8.2. Potential Application of RSM and ANNs in Addition to the Food Process

In addition to their applications in food processing, both RSM and ANNs have found potential applications in other areas of the food industry, including food quality, food safety, and food texture characteristics. Certifying the varieties and origins of extra virgin olive oil presents a significant challenge due to various factors influencing its properties, such as olive varieties, climatic conditions, and variations in production and storage methods. In a previous study, the application of an ANN successfully analyzed extensive datasets, achieving remarkable accuracy rates and exceeding 99% in certain instances [106]. Artificial intelligence (AI), when integrated with diverse data sources, is emerging as a transformative technology capable of swiftly addressing food safety concerns. Recent research has indicated that specific AI techniques may be more suitable for certain food safety applications than others [107]. The sensory attributes of apples, including their flavor, texture, color, and mouthfeel, are significantly affected by storage temperatures. To predict shelf life, prior research employed both the Partial Least Squares (PLS) and ANN techniques. The results demonstrate that lower storage temperatures are more effective in preserving apple color, pulp firmness, and the release of volatile compounds [108]. Furthermore, the ANN model, renowned for its nonlinear capabilities, proves to be a valuable and effective tool for predicting the shelf life of apples under both temperature settings. The results demonstrate that the integrated use of RSM and ANNs not only improves the accuracy of predictive models, as evidenced by higher R² and lower RMSE values, but also offers a more sustainable and efficient approach to food processing, which has not been previously documented in the literature.

## 9. Conclusions and Prospects

This review has extensively explored the application of multivariate methods, particularly Response Surface Methodology (RSM) and Artificial Neural Networks (ANNs), in optimizing the recovery of high-added-value compounds in the food industry. Our findings underscore these methods’ significant role in enhancing the efficiency, accuracy, and sustainability of food processing techniques. The superior predictive capabilities of ANNs, as evidenced by higher R² and lower RMSE values compared to RSM, highlight its potential as a leading tool in complex, nonlinear problem-solving scenarios within the food industry. This advantage is critical in addressing the dynamic and multifaceted challenges of food processing, ranging from nutrient preservation to the optimization of sensory qualities.

Furthermore, the application of these methods extends beyond mere technical enhancements; they represent a significant step towards more sustainable food processing practices. By optimizing resource utilization and reducing waste, these methods contribute to a more eco-friendly and economically viable food industry. This aspect is particularly pertinent in the current global context, where sustainability is increasingly becoming a priority. Looking forward, the integration of these methods with emerging technologies like AI and machine learning presents exciting opportunities for further advancements in the field. However, this also brings forth challenges such as scalability, the need for extensive datasets, and the balance between technological innovation and environmental responsibility. This optimized approach can be applied to various sectors within the food industry, including the extraction of bioactive compounds, enhancing the nutritional profile of food products, and improving shelf life, ultimately leading to cost savings and better product quality.

In conclusion, our study reaffirms the relevance and potential of RSM and ANNs in the food industry, not only as tools for optimization but also as catalysts for innovation and sustainability. While these methods have shown promising results, there remains ample scope for future research that could explore the application of this integrated optimization approach to other food matrices or extend the methodology to scale-up studies, ensuring its applicability in industrial settings. Additionally, further experimentation could involve the integration of emerging technologies such as machine learning algorithms to further enhance their predictive accuracy and process optimization. The future of food processing, guided by these advanced methodologies, looks toward a more efficient, sustainable, and health-conscious industry.

## Figures and Tables

**Table 2:** Antioxidant compounds are used to optimize antioxidant properties from food processing using the Artificial Neural Networks (ANNs) or Response Surface Methodology (RSM).

Target Compounds	Type of Multifactorial Design	Source	Optimal Conditions	Reference
Antioxidant activity	RSM	Pineapple peel waste	Solvent to substrate ratio of 20:1 mL/g, microwave power of 600 W, and extraction time 40 min	[69]
Anthocyanins	RSM	*Purple-heart* Radish	Optimized conditions: 25.0 g/100 g cellulase dosage, 60 mL/100 mL ethanol concentration, pH of 4.0, temperature of 50 °C, extraction time of 7.15 min, 80 mesh particle size, and 60:1 mL/g liquid-to-material ratio	[70]
Total phenolic content, total flavonoid content, and antioxidant activity	RSM	Buckwheat (*Fagopyrum esculentum Moench*)	Sample to solvent ratio of 1:35, extraction time of 49.045 min	[71]
Polyphenols extraction with ultrasound-assisted	RSM and ANN	Domestic garlic (*Allium sativum* L.)	Extraction time of 13.5 min, temperature of 59.0 °C, methanol concentration of 71.0% and solvent-to-solid ratio of 20.0 mL/g	[14]
Polyphenols	RSM and ANN	Defatted wheat germ	ANN showed minor advantage in fitting quality; RSM provided further insights via influence analysis. Optimization conditions for RSM were ethanol concentration of 58.0%, extraction time of 14.5 min, liquid-solid ratio of 25 mL/g, and irradiation power of 800 W; optimization conditions for ANN were ethanol concentration of 50.0%, extraction time of 15 min, liquid-solid ratio of 25 mL/g, and irradiation power of 400 W.	[72]
Polysaccharides and polyphenols	RSM and ANN	*Schisandra Chinensis*	ANN displayed better prediction capability than RSM. Optimal high-pressure conditions in ANN were liquid-to-material ratio of 87.1 mL/g, pressure of 4.8 MPa, temperature of 60.9 °C, and time of 16.0 min.	[73]
Carotenoid compounds	RSM	Watermelon pulp	6.025 g/g of Span 20/pulp sample-(S), 8.827 of glycerol/S, temperature of 38.75 °C, and time of 18.75 min (Extraction-1); 1.13 g/g of SMP/S, 7.53 g/g of Salt/S, 66.25 °C and 46.3 min (Extraction-2).	[74]
Carotenoids recovery	RSM	Carrot waste	Ultrasonic power of 213 W, extraction time of 36.0 min, and temperature of 44.6 °C	[75]
Carotenoid extraction	RSM	Cashew apple	38% acetone, 30% ethanol, and 32% petroleum ether for conventional extraction (CE); 44% acetone and 56% methanol for ultrasonic-assisted extraction (UAE)	[76]
Carotenoid composition	RSM	Paprika oleoresin	Static time of 28 min, extraction temperature of 78 °C, and 2 cycles for accelerated solvent extraction (ASE)	[77]

## Data Availability

Data sharing is not applicable.
